# Antioxidant Properties of Albumin and Diseases Related to Obstetrics and Gynecology

**DOI:** 10.3390/antiox14010055

**Published:** 2025-01-06

**Authors:** Kazushi Watanabe, Hiroyuki Kinoshita, Tomohito Okamoto, Kazumasa Sugiura, Shingo Kawashima, Tetsuro Kimura

**Affiliations:** 1 Department of Obstetrics and Gynecology, Aichi Medical University School of Medicine, Aichi 480-1195, Japan; ajipingpong@gmail.com (T.O.); sugiura.kazumasa.489@mail.aichi-med-u.ac.jp (K.S.); 2 Department of Dental Anesthesiology, Tokushima University Graduate School of Biomedical Sciences, Tokushima 770-8501, Japan; 3 Departments of Anesthesiology and Intensive Care, Hamamatsu University School of Medicine, Hamamatsu 431-3192, Japan; shingogo@hama-med.ac.jp (S.K.); t-kimura@hama-med.ac.jp (T.K.)

**Keywords:** albumin, antioxidant, preeclampsia

## Abstract

Albumin, the most abundant protein, contributes significantly to various physiological processes, indicating its multifunctional properties. It has drawn the attention of scientists and physicians because of its primary role in maintaining osmotic pressure and involvement in transporting numerous small molecules, including hormones, fatty acids, and drugs. A growing body of evidence has recently illustrated an additional aspect of albumin’s antioxidant properties. Therefore, based on recent research findings, this review article delves into the molecular and biochemical aspects of albumin’s antioxidative capabilities. We highlight the multifaceted significance of proteins in oxidative stress and their relation to pathologies in obstetrics and gynecology. In particular, we focused on preeclampsia, in which oxidative stress is closely involved in the pathogenesis, and renal dysfunction leads to increased albumin excretion into the urine, resulting in hypoalbuminemia. In addition, we discussed the role of albumin in preeclampsia pathogenesis, diagnosis, and patient prognosis. Understanding the antioxidant properties of albumin opens new avenues for therapeutic intervention and sheds light on novel strategies for combating preeclampsia associated with oxidative damage. In this study, we employed the PubMed database to search for articles that assessed the antioxidant properties of albumin, with a specific focus on obstetric diseases, particularly preeclampsia. The last update of the search was conducted in November 2024.

## 1. Introduction

Albumin is a multifunctional serum protein that maintains human physiology [1]. As the most abundant protein, its significant contributions to various physiological processes underscore its multifunctional nature [1]. Scientists and physicians have been drawn to albumin because of its primary role in maintaining osmotic pressure [2] and its involvement in transporting numerous small molecules [3,4,5,6], including hormones, fatty acids, and drugs [3,5]. A growing body of evidence has recently highlighted its functional and antioxidant properties [7,8,9,10,11,12]. Regarding the antioxidant properties of albumin, functions such as the neutralization of free radicals [1], protection of antioxidants [13,14,15], transport of antioxidants [16], and interaction with agents [17,18] have been identified.

The term “antioxidant” is synonymous with protection against oxidative stress, a phenomenon characterized by the excessive production of reactive oxygen species and free radicals that lead to cellular damage and tissue injury [19,20,21]. Many antioxidants act as scavengers of harmful species, thereby neutralizing their detrimental effects [1]. Despite being primarily recognized for its transport functions, albumin has emerged as a significant player in the body’s defense against oxidative stress [16,22,23,24].

This review article delves into the molecular and biochemical aspects of albumin’s antioxidative capabilities, drawing upon recent research findings. It highlights the multifaceted significance of proteins in the context of oxidative stress and their relationship to pathologies in obstetrics and gynecology. Providing a comprehensive overview of albumin’s characteristics, general functions, and its newfound role as an antioxidant has become increasingly critical. Within this context, we describe the structural features that enable albumin to engage in antioxidative activities, explore the mechanisms through which it combats oxidative stress, and discuss the potential implications for women’s health. Additionally, we examine the role of albumin in obstetric and gynecological diseases. In particular, we focused on preeclampsia, in which oxidative stress is closely involved in the pathogenesis, and renal dysfunction leads to increased albumin excretion into the urine, resulting in hypoalbuminemia. We discussed the role of albumin in preeclampsia pathogenesis, diagnosis, and patient prognosis.

In this study, we employed the PubMed database to search for articles that assessed the antioxidant properties of albumin, with a specific focus on obstetric diseases, particularly preeclampsia. The search utilized combinations of the following terms: “antioxidant albumin”, “oxidative stress”, “inflammation”, “preeclampsia”, “vascular endothelial dysfunction”, “renal dysfunction”, and “chronic kidney disease”. The last update of the search was conducted in November 2024.

## 2. Albumin’s Previously Known Characteristics, Functions, and Roles

Albumin is a vital and highly versatile protein found in the plasma of mammals, including humans. It is the most abundant plasma protein in the human circulatory system, with a molecular weight of approximately 66,000 Da, contributing to approximately 60% of the total plasma protein content [1,25]. Albumin is synthesized in the liver at up to 0.7 mg/h per gram (10–15 g/day) and secreted continuously without storage [11,26,27]. It is a critical regulator in many physiological and pathological aspects, such as colloid osmotic pressure, nutritional status, and inflammation [28,29,30,31]. Its kinetics depend on its synthesis, distribution within interstitial tissues and intravascular compartments, and excretion, indicating its significance in maintaining the colloid osmotic pressure of the blood, which is essential for proper fluid distribution between the bloodstream and tissues [29].

Albumin is a globular protein with a distinct three-dimensional structure consisting of a single polypeptide chain with multiple subdomains [4,7,11]. Their unique structures allow them to interact with various molecules, resulting in a wide range of functions. One of the primary roles is its action as a carrier or transporter targeted to variable substances, and it binds to a diverse array of small molecules, including fatty acids, hormones, bilirubin, drugs, and ions [4,32,33,34,35,36,37]. The albumin binding to these substances facilitates the appropriate and secure transportation of the albumin-conjugated complex into the bloodstream. This process determines the efficient delivery of molecules to target tissues and organs [3,27].

Albumin is critical in maintaining oncotic or colloid osmotic pressure in blood vessels [28,38,39,40]. The oncotic pressure gradient caused by albumin is essential for preventing excessive fluid leakage from the blood into the surrounding interstitial tissues, helping to maintain an appropriate blood volume and pressure. Therefore, albumin regulates the distribution of water, ions, and other molecules [41,42]. Its abundance, unique structure, and ability to transport various molecules make it indispensable for maintaining stable functions in the human body.

Albumin, with its diverse functions and roles, is indispensable for sustaining life. In conclusion, its multifaceted contributions underscore its significance in various biological processes, emphasizing its irreplaceable role in maintaining stable functions within the human body.

## 3. Albumin’s Antioxidant Properties

Previous studies have demonstrated the antioxidant properties of albumin, including its ability to neutralize harmful reactive oxygen species and free radicals, thereby protecting cells and tissues from oxidative stress. These findings may provide new insights into its significance in various pathophysiological conditions. This section summarizes previous studies and their results regarding human serum albumin as an antioxidant. Additionally, as shown in Table 1, it discusses the direct effects of albumin on oxidants and its indirect actions in supporting other antioxidants (Table 1). Furthermore, we address the specific origins of albumin beyond humans, such as bovine serum albumin.

### 3.1. Neutralization of Free Radicals by Albumin

Albumin directly or indirectly neutralizes reactive oxygen species and free radicals, reducing oxidative stress in living cells and tissues.

Albumin stabilizes hydroxyl radicals, a highly reactive radical, resulting in oxidative stress reduction in cells and tissues [16,22,43,44,45,46,47,48,49,58]. The reduced form of human serum albumin, also known as reduced human albumin (reduced albumin; human mercapto-albumin: HMA), directly reacts with hydroxyl radicals and is converted to an oxidized form (oxidized albumin; human non-mercapto-albumin: HNA) [8,11,24,43]. This process involves a hydroxyl radical neutralization caused by Cysteine-34 within human serum albumin as a critical cysteine residue for its function [1,43,50,62,63]. When free radicals, such as the hydroxyl radicals, are encountered, the Cysteine-34′s thiol group instantly reacts with them [43], indicating the mechanism highlighting the significance of Cysteine-34 in albumin’s role as a potent antioxidant in biological systems. In contrast, the counteraction of Cysteine-34 with radicals facilitates the conversion of HMA to HNA [16,59,64,65,66].

Albumin reacts with peroxyl radicals, resulting in the inactivation of the radicals [51,67]. Highly reactive peroxyl radicals induce lipid peroxidation and damage cell membranes and biological molecules, suggesting that albumin protects cells from these radicals. In addition, albumin neutralizes peroxyl radicals owing to its ability to bind hydrophobic molecules, such as fatty acids and pharmacological agents, as albumin-bound hydrophobic molecules are likely not utilized for lipid peroxidation processes.

Albumin also indirectly neutralizes highly reactive singlet oxygen, which mediates oxidative damage to biomolecules, including lipids, proteins, and DNA, through its ability to bind hydrophobic molecules, similar to its effect on peroxyl radicals [53].

Albumin’s antioxidative reactions involve scavenging superoxide radicals, thus mitigating oxidative damage. Additionally, albumin has been observed to inhibit NADPH oxidase, an enzyme responsible for generating superoxide in cells. By inhibiting membrane recruitment of the NADPH oxidase cytosolic submit p47phox, albumin further contributes to the reduction of superoxide production, helping to maintain a balance in oxidative processes [54,55]. Albumin demonstrates antioxidant effects by directly interacting with superoxide and by inhibiting the activity of NADPH oxidase, ultimately playing a protective role against oxidative stress.

Albumin acts as a potent antioxidant by directly neutralizing hydroxyl radicals and peroxyl radicals, inhibiting lipid peroxidation, and scavenging superoxide radicals. The critical cysteine residue, Cysteine-34, plays a key role in these antioxidative reactions. Additionally, albumin indirectly neutralizes singlet oxygen by binding hydrophobic molecules. Moreover, albumin inhibits NADPH oxidase, reducing superoxide production and contributing to the overall protection against oxidative stress in living cells and tissues.

### 3.2. Protection of Antioxidants by Albumin

Albumin plays a crucial role in protecting dietary antioxidants. Albumin stabilizes dietary antioxidants, including L-ascorbic acid (vitamin C), α-tocopherol (vitamin E), procyanidin B3, β-carotene, and astaxanthin, resulting in the prolongation of their antioxidant property. Indeed, ovalbumin counteracts oxygen-derived free radicals by binding to dietary antioxidants [13]. Albumin stabilizes (-)-epigallocatechin gallate, a component and antioxidant in green tea, in human serum [14]. In addition, bovine serum albumin stabilizes the antioxidant blueberry anthocyanins and enhances their antioxidant activity [15]. These results indicate the role of dietary antioxidants in oxygen-derived free radicals.

### 3.3. Transport of Antioxidants by Albumin

Albumin is responsible for transporting lipids and easily oxidizable antioxidants into the blood, allowing them to reach their destinations and exert their effects before undergoing oxidation. In the bloodstream, albumin binds to various substances, including fatty acids, bilirubin, bile acids, calcium, iron, copper, zinc, other cations, drugs, and tryptophan [16]. Albumin is a crucial ligand for free fatty acids, particularly polyunsaturated ones. This binding prevents peroxidation and reactive oxygen species formation. Lipid affinity also varies; for instance, proatherosclerotic lysophosphatidylcholine and lysophosphatidic acid exhibit a higher affinity for the oxidized form, whereas antiatherosclerotic derivatives of eicosapentaenoic and docosahexaenoic acids preferentially bind to the reduced albumin form [68]. It is essential to spotlight arachidonic acid and its derivatives to delve deeper into the intricate relationship. Arachidonic acid, a prominent polyunsaturated fatty acid, is a pre-cursor for synthesizing eicosanoids, which are active compounds. These eicosanoids, including prostaglandins, thromboxanes, and leukotrienes, play pivotal roles in various physiological processes, such as inflammation and immune response [69]. The dynamic interaction between albumin and arachidonic acid derivatives underscores a nuanced regulation of lipid metabolism and signaling pathways. Understanding these intricacies sheds light on the multifaceted nature of albumin’s role in maintaining the delicate balance between proatherosclerotic and antiatherosclerotic processes, offering valuable insights into the broader landscape of cardiovascular health.

In addition, albumin readily binds to metals in the blood. Notably, the binding properties of albumin demonstrate significant benefits, especially for metals, such as Cu^2+^ and Fe^3+^, which are highly pro-oxidative in their free forms. These metals interact with hydrogen peroxide in the free state, forming hydroxyl radicals with deleterious effects.

Through its binding properties, albumin may regulate the availability of these substrates and, among other actions, inhibit the pro-oxidative effects of metals and fatty acids [44].

Albumin serves as a crucial transporter for lipids and easily oxidizable antioxidants in the bloodstream, preventing peroxidation and reactive oxygen species formation. It binds various substances, including fatty acids, bilirubin, bile acids, and metals like Cu^2+^ and Fe^3+^, regulating their availability and inhibiting their pro-oxidative effects. The dynamic interaction between albumin and arachidonic acid derivatives highlights its nuanced role in lipid metabolism and signaling pathways, offering insights into cardiovascular health.

An important consideration is the redox state of albumin, which may differentially influence its interactions with various substances. For example, reduced albumin exhibits stronger binding affinity for Cu^2+^ and can transport nitric oxide, whereas oxidized albumin demonstrates a higher affinity for lipid peroxides. This dual behavior highlights the nuanced role of albumin in modulating oxidative stress. While the transport of lipid peroxides by oxidized albumin could potentially exacerbate oxidative stress, it may also serve as a protective mechanism by preventing their accumulation in vulnerable tissues. These contrasting effects underscore the complexity of albumin’s role in oxidative stress regulation and warrant further exploration.

### 3.4. Albumin Interaction with Agents

Albumin binds to numerous agents and facilitates their transport and distribution. This binding influences the metabolism and elimination of specific agents, mitigating harmful side effects from oxidation.

Quercetin, a polyphenol belonging to the flavonoid group, is a natural plant compound characterized by a yellow crystalline structure with a flavone backbone. It is naturally found in various foods, including citrus fruits, apples, onions, broccoli, kale, tea, red wine, and berries. Quercetin protects cells from oxidative stress and reduces the damage caused by reactive oxygen species, contributing to overall cellular health because of its potent antioxidant properties. Albumin binds quercetin and prevents its oxidation [17,18,60]. It has been reported that tea catechins bind to albumin, thereby enhancing its oxidative effect [61].

## 4. Albumin’s Role in Pathologies Related to Obstetrics and Gynecology

The following sections describe the role of albumin in pathologies related to obstetrics and gynecology. We focused on the role of hypoalbuminemia and oxidative stress in patients with preeclampsia.

### 4.1. Vascular Endothelial Dysfunction in Patients with Preeclampsia

Preeclampsia is one classification within hypertensive disorders of pregnancy. Preeclampsia is a pregnancy-related condition characterized by high blood pressure and damage to maternal organs or placenta [70]. Endothelial dysfunction is a fundamental pathological event in preeclampsia [71]. Endothelial injury reduces the vasodilatory effects caused by nitric oxide and prostacyclin and enhances the production of vasoconstrictor substances, including endothelin-1 and thromboxane, thereby leading to vasospasms. Therefore, placental vasospasm, which indicates a vasomotor imbalance between maternal and fetal blood flow, is a common feature of these disorders [72]. Using umbilical arteries obtained from pregnant women with hypertensive disorders of pregnancy, Okatani et al. showed that serotonin-induced constriction was enhanced in the arteries [73,74]. In addition, constriction is combined with impaired endothelial function, resulting in further decreased production of vasodilator substances, including nitric oxide and prostacyclin [73,74,75]. In addition, they identified maternal endothelial dysfunction in pregnant women with preeclampsia by evaluating the brachial artery vasodilation rate using the shear stress test (flow-mediated dilation: FMD) as an indicator of vascular endothelial function [76,77]. FMD in the brachial arteries, assessed by a high-resolution ultrasound, is widely used to measure endothelial function [78]. The technique provokes the release of nitric oxide, resulting in vasodilation that can be quantitated as an index of vasomotor function. FMD during pregnancy was significantly decreased in pregnant women with preeclampsia compared with normal pregnant women [76,77].

Vascular endothelial dysfunction is pivotal in preeclampsia, causing impaired vasodilation and heightened vasoconstriction. This imbalance leads to placental vasospasm and compromised blood flow regulation. Preeclamptic patients exhibit enhanced serotonin-induced constriction and decreased production of vasodilator substances such as nitric oxide and prostacyclin. Maternal endothelial dysfunction in preeclampsia has been identified through assessments like the FMD of the brachial artery, which significantly decreases compared to normal pregnancies.

### 4.2. Oxidative Stress in Patients with Preeclampsia

**Figure 1:**
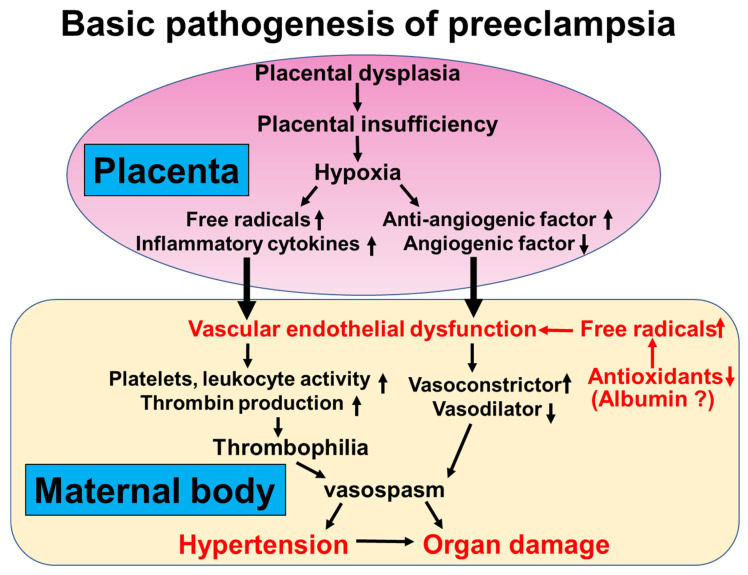
Basic pathogenesis of preeclampsia.

Pregnancy increases oxidative stress, a phenomenon generated by a normal systemic inflammatory response that leads to high levels of circulating reactive oxygen species. High maternal and fetal oxygen demands during pregnancy increase oxidative metabolism and promote free radical generation [79]. The primary source of reactive oxygen species during pregnancy is the placenta, which regulates this condition [80]. Placental insufficiency because of the inadequate remodeling of the maternal vasculature perfusing the intervillous space is considered the etiology of preeclampsia [81,82,83]. This condition mediates hypoxia in combination with the reoxygenation of the uteroplacental vessels. Subsequently, free radicals are released from the poorly perfused fetoplacental unit, initiating oxidative stress and further increasing oxidative stress in placental cells [84,85,86]. The plasma membranes of circulating blood cells are oxidized when they pass through the ischemic placenta, contributing to oxidative stress propagation in circumflex tissues [85,86,87] (Figure 1). Kimura et al. identified hypoxic changes in placental trophoblast cells in women with preeclampsia through immunohistochemical analysis for hypoxia-induced factor-1α, a marker of hypoxia status [88]. Additionally, oxidative DNA damage in placental trophoblast cells was observed in women with preeclampsia by conducting immunohistochemical analysis for 8-hydroxy-2′-deoxyguanosine and redox factor-1, which serve as markers of oxidative DNA damage and repair functions [88,89]. The superoxide produced by the placental mitochondria appears to be a significant source of oxidative stress, contributing to an overall increase in maternal blood and placental lipid peroxidation in women [90,91,92,93]. They suggest that the increased oxygen free radicals originate from the placenta in women with preeclampsia, as these free radicals decrease after delivery [89]. They also documented increased oxidant generation during the metabolism of hypoxanthine to uric acid in early-onset preeclampsia [94]. However, increased oxidative stress is counterbalanced by increased antioxidant synthesis [83]. Furthermore, their investigation revealed an elevation in maternal oxidative stress and fetal oxidative stress in cases of fetal growth restriction (FGR) [95]. They assessed the concentrations of derivatives of reactive oxygen metabolites (d-ROMs) as indicators of oxygen free radicals in the umbilical artery and vein. Significantly increased levels of d-ROMs were observed in the umbilical artery and vein in preeclamptic women with FGR, whereas no such increase was noted in those without FGR. Additionally, concentrations of d-ROMs in the umbilical artery were notably higher than those in the umbilical vein in preeclamptic women with FGR, a distinction not observed in preeclamptic women without FGR. These findings suggest that oxidative stress occurring in the maternal body also impacts the fetus in preeclamptic women with FGR.

Evidence indicates that antioxidant enzyme levels increase as reactive oxygen species production increases, maintaining a balance between antioxidants and reactive oxygen species [96,97]. The first line of defense against reactive oxygen species consists of antioxidant enzymes, including superoxide dismutase, glutathione peroxidase, and catalase, which metabolize these reactive species into innocuous by-products [98]. The non-enzymatic antioxidants represent the second line of defense against reactive oxygen species and include low-molecular-weight compounds, such as vitamins C and E, α tocopherol, β-carotene, lipoic acid, ubiquinone, carotenoids, uric acid, and glutathione [99]. These molecules reduce and rapidly inactivate the radicals and oxidants [100]. Other scavengers and metal chelators, such as ceruloplasmin, albumin, ferritin, and lactoferrin, contribute to maintaining antioxidant status [98]. When oxidative stress exceeds the antioxidant defense in the placenta, oxidative damage can propagate to distal tissues.

Oxidative stress in preeclampsia is exacerbated by increased maternal and fetal oxygen demands, leading to heightened reactive oxygen species production primarily from the placenta. This oxidative imbalance is further compounded by inadequate placental remodeling and hypoxia–reoxygenation cycles. Antioxidant enzyme levels rise to counteract oxidative stress, but when overwhelmed, oxidative damage can extend to distant tissues. Additionally, oxidative stress is exacerbated in the case of preeclampsia with FGR. Oxidative stress markers are elevated in both maternal and fetal circulations, indicating a systemic impact. Antioxidant defense mechanisms, including enzymatic and non-enzymatic pathways, play crucial roles in maintaining redox balance.

### 4.3. Oxidative Stress in Combination with Vascular Endothelial Dysfunction in Patients with Preeclampsia

The hallmark of the condition is endothelial cell damage in patients with preeclampsia [101,102]. The activation and dysfunction of the maternal endothelium in preeclampsia are mediated, in part, through the release of placentally derived factors [103]. Hypoxic trophoblasts release antiangiogenic cytokines and free radicals into the maternal circulation (Figure 1). Oxidative stress from reactive oxygen species causes vascular endothelial dysfunction in patients with preeclampsia. Wakatsuki et al. demonstrated that the addition of reactive oxygen species and oxidized low-density lipoproteins to umbilical arteries impairs the endothelial production of nitric oxide and prostacyclin, making them contractile, proving that oxidative stress is involved in the impairment of endothelial function [104,105]. They also measured the levels of reactive oxygen species metabolites and antioxidants in the blood of pregnant women. They found that oxidative stress is enhanced in women with preeclampsia, not by changes in antioxidants but by increased production of reactive oxygen species metabolites [76]. In addition, they documented that the endothelial function of pregnant women with hypertensive disorders of pregnancy was markedly reduced, and one of the causes of this was oxidative stress as determined by FMD in pregnant women [76]. They demonstrated a negative correlation between FMD and d-ROM concentrations in pregnant women [76]. Furthermore, they also demonstrated a negative correlation between FMD and d-ROMs concentrations, which was observed in pregnant women with preeclampsia but not in normal pregnant women [77]. Maternal endothelial function is likely to mediate a systemic increase in oxygen-derived free radicals in women [76,77,94], resulting in oxidative stress and maternal morbidity.

Maternal endothelial dysfunction in preeclampsia, driven by placentally derived factors, leads to oxidative stress and impaired production of nitric oxide and prostacyclin. Increased levels of reactive oxygen species metabolites contribute to endothelial dysfunction, exacerbating vascular complications in pregnant women with hypertensive disorders. This systemic increase in oxygen-derived free radicals underscores the role of oxidative stress in maternal morbidity associated with preeclampsia.

### 4.4. Antioxidant Effect of Albumin in Patients with Preeclampsia

Recently, albumin has garnered attention as an antioxidant [7,8,9,10,11,12]. Reports on albumin as an antioxidant in patients with preeclampsia are still limited but are gradually emerging. Indeed, it is critical to note that elevated free radical activity and oxidative stress contribute to the pathophysiology of preeclampsia [76,83].

Albumin is a scavenger of free radicals, and reactive oxygen species has been implicated in the pathogenesis of preeclampsia. Through its electron-donating ability and neutralization of oxidative species, albumin helps maintain redox homeostasis, protecting renal tissues from oxidative damage [106,107,108,109,110,111,112]. Antioxidant function is particularly relevant in preeclampsia-associated nephropathy, in which increased oxidative stress contributes to renal injury [113,114,115]. Albumin undergoing structural modifications by reactive oxygen species is vulnerable to a reduction in its binding capacity to transition metals and the formation of ischemia-modified albumin, a sensitive marker in conditions of oxidative stress related to ischemia–reperfusion [52,116,117]. Within the hypoxic environment, typical of the preeclamptic placenta, the N-terminal region of albumin exhibits a reduced capacity to bind to cobalt, leading to the formation of chemically altered albumin, referred to as ischemia-modified albumin [118,119]. In contrast, ischemia-modified albumin is a marker of preeclampsia [120,121,122,123,124,125,126]. The negative correlation between albumin and ischemia-modified albumin observed in preeclampsia indicates the role of albumin as an oxidative stress scavenger [120,121].

Kinoshita et al. suggested that human serum albumin, as well as that derived from bovine serum, reduces oxidative stress by inhibiting reduced nicotinamide adenine dinucleotide phosphate (NADPH) oxidase in human vascular smooth muscle [10]. Indeed, in human vascular smooth muscle cells, albumin reduces superoxide levels by inhibiting membrane recruitment of the NADPH oxidase cytosolic subunit p47phox, a critical enzyme involved in oxygen-derived free radical production in cardiovascular diseases [10,54,55]. In women with preeclampsia, the onset of proteinuria often coincides with a rapid increase in protein excretion, leading to hypoalbuminemia [10,56,57,113,127]. The relationship between hypoalbuminemia and the heightened production of oxygen-derived free radicals remains unclear, although some studies have suggested a potential association [10]. Indeed, they suggested that serum albumin reduces oxidative stress by inhibiting NADPH oxidase in human vascular smooth muscle [10]. Notably, decreased serum albumin levels are inversely related to oxidative stress and positively associated with preserved endothelial function in pregnant women [10]. Therefore, reduced serum albumin levels predict the increased production of oxygen-derived free radicals, resulting in a maternal vascular endothelial function in parturients with preeclampsia [56].

Previous studies have demonstrated that albumin modulates inflammatory responses, another component of the pathophysiology of preeclampsia [128]. Accordingly, by reducing inflammation in the disease state, albumin may also indirectly limit oxidative stress, given the interconnectedness of inflammation and oxidative stress.

Albumin serves as an antioxidant in preeclampsia, scavenging free radicals and maintaining redox homeostasis. Additionally, albumin’s modulation of inflammation may indirectly mitigate oxidative stress in the context of preeclampsia.

### 4.5. Mechanistic Insight into Hypoalbuminemia in Patients with Preeclampsia

Women with preeclampsia exhibit elevated urinary albumin excretion, leading to hypoalbuminemia. However, the precise mechanism underlying the increased renal albumin excretion remains unclear. The specificity of renal tissue damage in women with preeclampsia is not well understood. A defense mechanism exists in the kidneys for the urinary excretion of albumin. The endothelial glycocalyx layer, situated on the luminal surface of all endothelial cells, contributes to the permeability barrier formed by the vessel wall [129,130]. The glycocalyx is the initial barrier in the defense mechanism against urinary albumin excretion. The adherent structures include proteoglycans, glycoproteins, and glycolipids. Proteoglycans comprise core proteins, such as syndecans, glypicans, and biglycan, with covalently bound glycosaminoglycan side chains, such as heparan sulfate and chondroitin sulfate. Proteoglycans covalently bind to the endothelial cell membrane and adhere to glycosaminoglycans, including long hyaluronan chains [131,132]. The second barrier is the podocytes. Podocytes are located on the outer surface of the glomerular basement membrane of the kidneys. Podocytes regularly interlock with foot projections of adjacent podocytes. The slit membrane stretched between the foot processes and served as an albumin barrier [57,133]. When these barriers are impaired, albumin is excreted in the tubules.

Furthermore, leaked albumin is reabsorbed in the renal tubules, and only a minimal amount is excreted in the urine. Pathological albumin excretion into the urine occurs when the defense mechanisms against albumin excretion are impaired. Asai et al. suggest that renal impairment in the glomerular endothelial glycocalyx, podocytes, and tubulointerstitium occurs in women with preeclampsia and proteinuria [57,112,113].

In contrast, in women with preeclampsia, urinary albumin excretion decreases rapidly, and postpartum renal dysfunction improves dramatically. However, the changes in the pathogenesis of renal injury during the postpartum period are unknown and have not yet been reported. We examined the trends in urinary albumin excretion and renal dysfunction postpartum. We found that proteinuria decreased to the level observed in healthy pregnant women at one month postpartum in women with preeclampsia. However, urinary albumin excretion was higher in patients with preeclampsia than in normal parturients. We further examined the sites of kidney damage at one month postpartum in women with preeclampsia. Endothelial glycocalyx and podocyte injuries remained residual in the patients, whereas the coexisting tubulointerstitial injury improved. We speculated that postpartum urinary protein excretion decreased because of improved tubular function, but endothelial glycocalyx and glomerular podocyte injuries persisted. Thus, albumin excretion persisted. Women with a history of preeclampsia exhibit a higher incidence of future chronic kidney disease [134,135]. Proteinuria induces nephrotoxic injury via various pathways. For example, albumin reabsorbed by the proximal tubular epithelial cells can cause damage, resulting in a pro-inflammatory phenotype with the production of cytokines and chemokines [114,115]. Persistent urinary albumin excretion may lead to recurrent tubular damage and progression to chronic kidney disease. The involvement of oxidative stress in hypoalbuminemia remains controversial.

In women with preeclampsia, elevated urinary albumin excretion leads to hypoalbuminemia, which is linked to damage in the glomerular endothelial glycocalyx, podocytes, and tubuleinterstitium. While urinary protein excretion improves postpartum, the ongoing damage to the endothelial glycocalyx and podocytes continues to contribute to low-level albumin excretion, raising the long-term risk of chronic kidney disease in women with preeclampsia.

### 4.6. Mechanism Between Hypoalbuminemia and Inflammation in Patients with Preeclampsia

**Figure 2:**
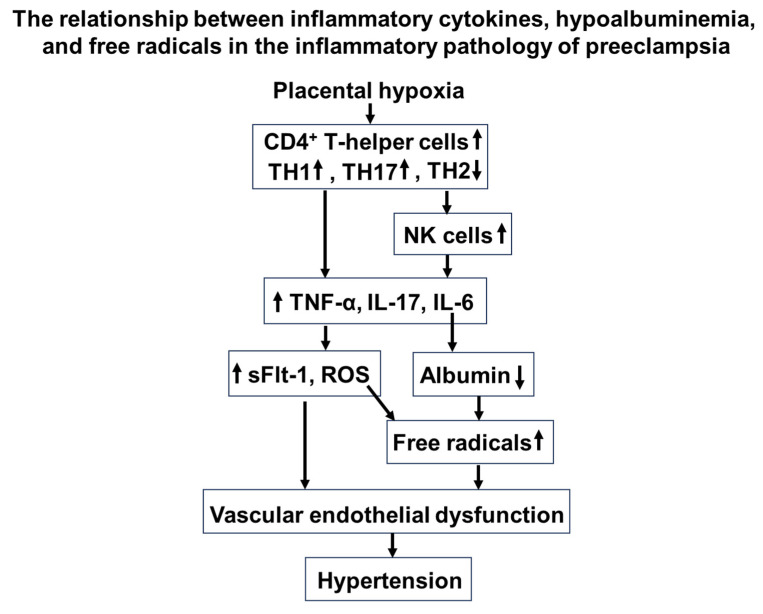
The relationship between inflammatory cytokines, hypoalbuminemia, and free radicals in the inflammatory pathology of preeclampsia.

The exact cause of preeclampsia is not fully understood, but there is a significant association with an inflammatory state. The inflammatory response is thought to play a crucial role in the development and progression of preeclampsia [136,137,138]. During pregnancy, the maternal immune system undergoes complex changes to tolerate the presence of the developing fetus [138,139]. However, this immunological tolerance is disrupted in preeclampsia, leading to an exaggerated inflammatory response [138,140]. This inflammatory state is characterized by the release of pro-inflammatory cytokines, chemokines, and other immune mediators [141]. The immune dysregulation in preeclampsia contributes to endothelial dysfunction, a key feature of the condition. Endothelial cells line blood vessels and play a vital role in regulating blood flow. In preeclampsia, the inflammatory response adversely affects the function of these endothelial cells, leading to vasoconstriction and reduced blood flow to organs. The release of inflammatory substances also contributes to oxidative stress, causing damage to cells and tissues [142]. This oxidative stress further exacerbates endothelial dysfunction and disrupts the balance between vasodilation and vasoconstriction. The compromised vascular function contributes to the hallmark symptoms of preeclampsia, such as hypertension and organ damage. Overall, the intricate interplay between immune dysregulation, endothelial dysfunction, and oxidative stress creates a milieu conducive to the development of preeclampsia (Figure 2).

In the context of an inflammatory state, hypoalbuminemia can occur because of several mechanisms related to the altered synthesis and metabolism of albumin. Inflammation can lead to a suppression of albumin synthesis in the liver [43]. Cytokines, particularly interleukin-6 (IL-6), are key mediators of this response. Elevated levels of IL-6 inhibit albumin production, contributing to decreased circulating albumin levels [1,143]. The inflammatory state induces an increase in the catabolism of albumin [1,43]. This increase may be mediated by various proteolytic enzymes activated during inflammation, leading to a shorter half-life of albumin in the circulation. Inflammatory conditions can cause a shift in the distribution of albumin between the intravascular and extravascular compartments [43]. Increased capillary permeability, often observed in inflammation, can lead to the albumin leakage from the bloodstream into surrounding tissues, further reducing circulating levels [43,144,145] (Figure 2).

Placental hypoxia alters the population of CD4^+^ T helper cells, promoting NK cytolytic polarization and increasing inflammatory cytokine levels. These elevated cytokines further suppress the production of the antioxidant albumin, leading to reduced albumin levels. Consequently, the diminished antioxidant defense against reactive oxygen species contributes to the progression of preeclampsia pathology through vascular endothelial dysfunction (Figure 2).

Thus, hypoalbuminemia in preeclampsia is driven by both reduced albumin production and increased catabolism, compounded by renal impairment leading to heightened albumin excretion. The inflammatory response, therefore, plays a central role in both the pathogenesis of preeclampsia and the development of hypoalbuminemia.

### 4.7. Relationship Between Women with Previous Preeclampsia and Future Development of Chronic Kidney Disease Development

Pregnant women who experience preeclampsia and subsequently develop proteinuria often exhibit a rapid increase in protein excretion, particularly during pregnancy. The heightened urinary excretion of albumin, the protein component of urine, is a consequence of renal glomerular and tubular damage in women with preeclampsia, leading to hypoalbuminemia [56,57,113,127]. The reduced levels of blood albumin, an antioxidant factor, contribute to increased oxidative stress, causing maternal vascular damage, elevated blood pressure, and associated organ damage [56,57,113,127]. Considering the potential impact of hypoalbuminemia, albumin replacement therapy emerges as a prospective treatment for pregnant women with preeclampsia. However, as of now, no conclusive results have been reported. In cases where preeclampsia develops and renal impairment occurs, albumin replacement is swiftly excreted in the urine, raising uncertainties about its efficacy. A pregnant woman with a history of proteinuria-associated preeclampsia in a previous pregnancy may benefit from preventive measures against preeclampsia, especially if hypoproteinemia is observed prior to the onset proteinuria in such cases. Future research should focus on preventing preeclampsia in pregnant women with subclinical hypoalbuminemia who have not yet manifested renal damage.

Conversely, the condition of heavy proteinuria in pregnant women with preeclampsia closely resembles that of individuals with chronic kidney disease, putting them at a high risk of developing chronic kidney disease in the future [134,135]. While many women experience a rapid decline in protein and albumin excretion post-childbirth, some may retain residual proteinuria, indicating a potential risk for future chronic kidney disease. Specifically, a urinary Protein/Creatinine ratio of 0.15 g/g Creatinine or higher at any time or a urinary Albumin/Creatinine ratio of 30 mg/g Creatinine or higher at 12 weeks postpartum, when the effects of pregnancy have subsided, suggests residual renal impairment and a suspected risk of future chronic kidney disease development. Monitoring urinary protein levels, especially the Protein/Creatinine or Albumin/Creatinine ratio, is essential for identifying women at risk for long-term kidney damage. Early detection, ongoing nephrological follow-up, and thorough evaluation by a nephrologist are crucial for preventing progression to chronic kidney disease in these patients.

## 5. Conclusions

Preeclampsia enhances the levels of reactive oxygen species, free radicals, and inflammation activity, resulting in vascular endothelial dysfunction. Currently, low-dose aspirin therapy [146,147,148] and statin therapy [149,150] are the most promising approaches for preventing preeclampsia. Recently, the antioxidant properties of albumin have gained attention, promoting our focus on its role in the pathogenesis of preeclampsia. Preeclampsia involves oxidative stress as an etiological factor and leads to hypoalbuminemia owing to urinary protein excretion. Furthermore, the inflammation associated with preeclampsia elevates cytokine levels, suppressing albumin production and exacerbating hypoalbuminemia. This hypoalbuminemia, in turn, increases oxidative stress, contributes to vascular endothelial dysfunction, and underlines the fundamental pathophysiology of preeclampsia. Potential treatment strategies involving albumin for preeclampsia include albumin replacement and prevention of renal excretion. However, replacement therapy may have limited efficacy in cases with significant urinary albumin loss, becoming beneficial only if renal albumin excretion is effectively suppressed. Pregnant women with a history of proteinuria-associated preeclampsia may benefit from preventive measures, particularly if hypoproteinemia is observed prior to the onset proteinuria. At present, dietary modifications, such as restricting excessive calorie and salt intake, remain the only evidence-based intervention for renal protection in preeclampsia. Further research is essential to elucidate the relationship between preeclampsia and hypoalbuminemia. Additionally, women with a history of preeclampsia face a heightened risk of developing chronic kidney disease later in the future. Persistent urinary albumin secretion after delivery may exacerbate renal damage, potentially progressing to chronic kidney disease. Understanding the antioxidant properties of albumin not only provides new insights into the pathophysiology of preeclampsia but also opens avenues for innovative therapeutic strategies targeting oxidative damage in obstetric and gynecological disorders.

## Figures and Tables

**Table 1:** Albumin’s antioxidant properties.

Direct Effects		
Target Oxidants	Roles	References
Hydroxyl radical	Stabilization, Neutralization	[8,11,16,24,25,43,44,45,46,47,48,49,50,51,52]
Peroxyl radicals	Inactivation	[8,11,16,25,51]
Singlet oxygen	Inactivation	[53]
Superoxide	NADPH oxidase inhibition	[8,10,11,25,52,54,55,56,57]
**Indirect Effects**		
**Target Substances**	**Roles**	**References**
L-ascorbic acid	Stabilization, Protection	[13]
α-Tocopherol	Stabilization, Protection	[11,13]
Procyanidin B3	Stabilization, Protection	[13]
β-Carotene	Stabilization, Protection	[13]
Astaxanthin	Stabilization, Protection	[13]
(-)-Epigallocatechin gallate	Stabilization, Protection	[14]
Anthocyanins	Stabilization, Protection	[15]
Fatty acids, Bilirubin, Bile acids, Calcium, drugs, and Tryptophan, etc.	Binding to prevent peroxidation and reactive oxygen species formation of the substances	[8,11,16,43,53]
Metals, including Cu^2+^, Fe^2+^, Fe^3+^	Binding to prevent hydroxyl radical production	[8,11,16,43,44,58,59]
Quercetin and Catechin	Binding to enhance antioxidative effects of the substances	[17,18,60,61]
